# Meta-analysis of haematoma volume, haematoma expansion and mortality in intracerebral haemorrhage associated with oral anticoagulant use

**DOI:** 10.1007/s00415-019-09536-1

**Published:** 2019-09-20

**Authors:** David J. Seiffge, Martina B. Goeldlin, Turgut Tatlisumak, Philippe Lyrer, Urs Fischer, Stefan T. Engelter, David J. Werring

**Affiliations:** 1 Stroke Research Centre, Institute of Neurology, University College London, Russell Square House, 10 Russell Square, London, UK; 2 Stroke Center and Neurology, Department of Clinical Research, University Hospital and University, Basel, Switzerland; 3 Department of Neurology, Inselspital, Bern University Hospital, University of Bern, Bern, Switzerland; 4 University Institute for Diagnostic and Interventional Neuroradiology, Inselspital, Bern University Hospital, University of Bern, Bern, Switzerland; 5 Department of Clinical Neuroscience/Neurology, Institute of Neurosciences and Physiology, Sahlgrenska Academy at University of Gothenburg, Gothenburg, Sweden; 6 Department of Neurology, Sahlgrenska University Hospital, Gothenburg, Sweden; 7 Neurorehabilitation Unit, University Center for Medicine of Aging and Rehabilitation Basel, Felix Platter Hospital, University of Basel, Basel, Switzerland

**Keywords:** Oral anticoagulants, Intracerebral haemorrhage, Haematoma volume, Mortality, Haematoma expansion

## Abstract

**Objective:**

To obtain precise estimates of age, haematoma volume, secondary haematoma expansion (HE) and mortality for patients with intracerebral haemorrhage (ICH) taking oral anticoagulants [Vitamin K antagonists (VKA-ICH) or non-Vitamin K antagonist oral anticoagulants (NOAC-ICH)] and those not taking oral anticoagulants (non-OAC ICH) at ICH symptom onset.

**Methods:**

We conducted a systematic review and meta-analysis of studies comparing VKA-ICH or NOAC-ICH or both with non-OAC ICH. Primary outcomes were haematoma volume (in ml), HE, and mortality (in-hospital and 3-month). We calculated odds ratios (ORs) using the Mantel–Haenszel random-effects method and corresponding 95% confidence intervals (95%CI) and determined the mean ICH volume difference.

**Results:**

We identified 19 studies including data from 16,546 patients with VKA-ICH and 128,561 patients with non-OAC ICH. Only 2 studies reported data on 4943 patients with NOAC-ICH. Patients with VKA-ICH were significantly older than patients with non-OAC ICH (mean age difference: 5.55 years, 95%CI 4.03–7.07, *p* < 0.0001, *I*^2^ = 92%, *p* < 0.001). Haematoma volume was significantly larger in VKA-ICH with a mean difference of 9.66 ml (95%CI 6.24–13.07 ml, *p* < 0.00001; *I*^2^ = 42%, *p* = 0.05). HE occurred significantly more often in VKA-ICH (OR 2.96, 95%CI 1.74–4.97, *p* < 0.00001; *I*^2^ = 65%). VKA-ICH was associated with significantly higher in-hospital mortality (VKA-ICH: 32.8% vs. non-OAC ICH: 22.4%; OR 1.83, 95%CI 1.61–2.07, *p* < 0.00001, *I*^2^ = 20%, *p* = 0.27) and 3-month mortality (VKA-ICH: 47.1% vs. non-OAC ICH: 25.5%; OR 2.24, 95%CI 1.52–3.31, *p* < 0.00001, *I*^2^ = 71%, *p* = 0.001). We did not find sufficient data for a meta-analysis comparing NOAC-ICH and non-OAC-ICH.

**Conclusion:**

This meta-analysis confirms, refines and expands findings from prior studies. We provide precise estimates of key prognostic factors and outcomes for VKA-ICH, which has larger haematoma volume, increased rate of HE and higher mortality compared to non-OAC ICH. There are insufficient data on NOACs.

**Electronic supplementary material:**

The online version of this article (10.1007/s00415-019-09536-1) contains supplementary material, which is available to authorized users.

## Background

Intracerebral haemorrhage (ICH) is a devastating form of stroke with high mortality and morbidity [1]. Oral anticoagulants (OAC) are beneficial in the prevention of ischaemic stroke and systemic embolism in patients with atrial fibrillation. Vitamin K antagonists (VKA) were the only option for OAC therapy for many years, but since 2010, direct thrombin inhibitors [2] (dabigatran) and factor Xa inhibitors [3] (apixaban, edoxaban and rivaroxaban)—termed non-vitamin K antagonist oral anticoagulants (NOAC)—have emerged as an alternative option.

Current knowledge about the influence of OAC on haematoma volume, secondary haematoma expansion (HE) and mortality in ICH is mainly based on data from small single centre studies conducted in the 1990s [4, 5] or early 2000s [6–8]. These studies found that ICH in patients on VKA therapy (VKA-ICH) was associated with larger haematoma volume and higher rates of HE and mortality compared to patients not taking OAC (non-OAC ICH). By contrast, more recent studies were less consistent regarding the influence of VKA: some did not find differences in ICH volume [7, 9, 10], HE [9, 10] or mortality [9]; others found that VKA influences haematoma volume in non-lobar but not lobar ICH location [11], or if the INR was supratherapeutic (>3.0) [12].

Since the introduction of NOACs, several single-center [13, 14] and multi-center [15–17] studies analysed differences between VKA-ICH and ICH in patients on NOAC therapy (NOAC-ICH) with heterogeneous results.

We conducted a systematic review and meta-analysis of published studies to determine the most precise available estimates of age, haematoma volume, risk of HE and mortality for OAC-ICH (VKA-ICH or NOAC-ICH) compared to non-OAC ICH.

## Methods

The report was prepared with respect to the PRISMA recommendations [18]. The analysis was performed in 11/2018–01/2019 according to a pre-planned protocol developed by all investigators in 10/2018 (not published).

### Search strategy and inclusion/exclusion criteria

Two investigators (DJS and MBG) independently searched pubmed.gov/MEDLINE for relevant publications on 31 October 2018 and on 14 November 2018. We used the following search terms: [(oral anticoagulants OR Vitamin K antagonists or novel oral anticoagulants or direct oral anticoagulants or non-vitamin K antagonist oral anticoagulants) and (ICH) AND (mortality or volume)] including different spellings and common abbreviations (i.e. VKA, NOAC, DOAC, ICH).

We applied the following inclusion criteria:

- Original studies published in English,
- Comparing OAC-ICH (either VKA-ICH or NOAC-ICH or both) with non-OAC ICH.
- Recruiting patients from the same population (hospital-based or population-based studies).
- Reporting at least one of the following outcomes for both types of ICH (OAC-ICH and non-OAC ICH): mortality, haematoma volume or HE.

We applied the following exclusion criteria:

- Study with less than 10 participants with OAC-ICH.
- Studies reporting Matched cohorts rather than consecutive patients.

### Data collection

Two author (DJS and MBG) independently performed the literature research and screened all titles and abstracts for eligibility. We read the full text of articles potentially eligible for inclusion and independently extracted data on study design, demographics and outcomes. Disagreements were resolved by collegial discussion. We included the following outcomes.

- ICH volume: mean ICH volume in millilitre (ml) with standard deviation (SD); if median and interquartile range were provided, we extrapolated the mean and SD using a published formula and method [19].
- Haematoma expansion (HE), usually defined as haematoma volume increase of + 6 ml or + 33% between baseline and follow-up imaging.
- Mortality: either in-hospital or 3-month mortality.

VKA was defined as the use of warfarin, phenprocoumon, fluindione or acénocoumarol. NOAC was defined as the use of apixaban, dabigatran, edoxaban or rivaroxaban.

### Risk of bias

Two authors (DJS and MBG) independently assessed study quality and risk of bias using the scheme suggested by the Cochrane collaboration (“Tool to assess risk of bias in cohort studies”).

### Statistical analysis

We used Review Manager (RevMan) Version 5.3 (Copenhagen: The Nordic Cochrane Centre, The Cochrane Collaboration, 2014) statistical program package. Patients with VKA-ICH and NOAC-ICH were analysed separately and compared with patients with non-OAC ICH.

We used the Mantel–Haenszel random-effects method to compare mortality and HE and calculated odds ratios (OR) and corresponding 95% confidence intervals (CI). ICH volumes as continuous variable were pooled using the difference of means comparison based on the mean ICH volume reported in each study and the corresponding SD. We reported the mean difference of ICH volume (in ml) with corresponding SD. Heterogeneity was assessed using I^2^ statistics and displayed the results using forest plots.

## Results

The literature research identified 226 publications. After reading the titles and abstracts/full papers if applicable, we included 19 studies (see Table 1): 12 publications from observational single-centre studies [4, 5, 7–11, 20–24] (all tertiary care centres—two publications from the same centre reporting complementary outcome data [7, 8]); six observational multi-centre studies [6, 12, 25–28] [one population-based study with two publications [6, 12] reporting complementary outcome data and one national US Get-with-the-guidelines (GWTG) registry [25]]; and one sub-analysis of the placebo arm of a randomized controlled trial [29]. If there were multiple publications from the same study/centre, this study/centre only contributed data from one publication to each outcome. Altogether, the studies reported data of 150,270 patients with ICH of whom 16,546 (11.0%) had VKA-ICH, 4943 (3.3%) had NOAC-ICH and 128,761(85.7%) had non-OAC ICH. The large US GWTG study [25] contributed 141,311 (94.0%) patients but reported only outcome data for in-hospital mortality and not for haematoma volume, HE or 3-month mortality. The risk of bias for included studies was medium (Table 2).

**Table 1:** Included studies and their characteristics

Study		Study details						Available outcomes		
		Study period	OAC type	Number of patients	Follow-up	age VKA-ICH (mean ± SD^a^)	age non-OAC ICH (mean ± SD)	Mortality	ICH volume	HE
Single-center observational studies										
Radberg et al. [5]	1982–1986		VKA	VKA-ICH: 28, non-OAC ICH: 172	3 month	71 (range 48–78)	n/a	Yes	No	No
Neau et al. [4]	1984–1996		VKA	VKA-ICH: 79, non-OAC ICH: 127	In-hospital	69.4 (±9.1)	71.7 (±12.6)	Yes	Yes	No
Rosand et al. [8]	1994–2001		VKA	VKA-ICH: 102, non-OAC ICH: 333	3 month	75.7 (±8.4)	74 (±9.6)	Yes	No	No
Flibotte et al. [7]	1998–2002		VKA	VKA-ICH: 42, Non-OAC ICH: 141	In-hospital	n/a	n/a	No	Yes	Yes
Fric-Shamji et al. [22]	2002–2004		VKA	VKA-ICH: 65, non-OAC ICH: 250	In-hospital	71 (range 44–98)	64 (range 35–93)	Yes	Yes	Yes
Yamashita et al. [24]	2004–2009		VKA	VKA-ICH: 94, non-OAC ICH: 295	In-hospital	72 (±10.5)	66.2 (±12.7)	Yes	Yes	Yes
Horstmann et al. [9]	2009–2011		VKA	VKA-ICH: 51, non-OAC ICH: 155	3 month	76.7 (±6.9)	71 (±14.2)	Yes	Yes	Yes
Ma et al. [21]	2007–2012		VKA	VKA-ICH: 69, non-OAC ICH: 333	In-hospital	76 (±9.8)	72 (±14.1)	Yes	Yes	No
Dequatre-Ponchelle et al. [11]	2004–2009		VKA	VKA-ICH: 83, non-OAC ICH: 462	In-hospital	75 (±10.6)	68.7 (±16.4)	No	Yes	No
Curtze et al. [20]	2005–2010		VKA	VKA-ICH: 132, non-OAC ICH: 868	3 month	75.7 (±9.7)	66.7 (±15)	Yes	Yes	Yes
Von der Brelie et al. [10]	2011–2016		VKA, NOAC	VKA-ICH: 47, NOAC-ICH: 25, non-OAC ICH: 110	3 month	74.9 (±2.9)	68.7 (±14.4)	Yes	Yes	Yes
Roquer et al. [23]	2005–2015		VKA	VKA-ICH: 89, non-OAC ICH: 293	3-month	78.7 (±6.8)	72 (±16.4)	Yes	Yes	No
Multi-center observational studies										
Foerch et al. [26]		2003–2004	VKA	VKA-ICH: 208, non-OAC ICH: 1483	In-hospital	75.7 (±7)	70 (±14)	Yes	No	No
Flaherty et al. [6]		1998–2003	VKA	VKA-ICH: 190, non-OAC ICH: 851	In-hospital	74.8 (±11.7)	68.9 (±15.8)	Yes	No	No
Flaherty et al. [12]		2005	VKA	VKA-ICH: 51, non-OAC ICH: 207	In-hospital	n/a	n/a	No	Yes	No
Toyoda et al. [28]		1999–2003	VKA	VKA-ICH: 67, non-OAC ICH: 738	3-month	71 (±11)	65 (±13)	Yes	Yes	Yes
Romem et al. [27]		02–03/2004, 03–04/2007, 04–05/2010, 04–05/2013	VKA	VKA-ICH: 92, non-OAC ICH: 310	n/a	74.5 (±8.3)	71 (±14.2)	No	Yes	No
Inohara et al. [25]		2013–2016	VKA NOAC	VKA-ICH: 15,036, NOAC-ICH: 4918, Non-OAC ICH: 121,357	In-hospital	76.3 (±11.9)	67.7 (±17.1)	Yes	No	No
Subanalysis of a multi-center randomized controlled trial (placebo group)										
Cucchiara et al. [29]		2005	VKA	VKA-ICH: 21, non-OAC ICH: 282	3 month	75 (±7.9)	65 (±11.8)	Yes	Yes	Yes

*NOAC* apixaban, dabigatran and rivaroxaban

^a^Age: if median and IQR were provided, we estimated mean and SD using a previously published formula(19). VKA: Warfarin (except Horstmann et al. [9]: phenprocoumon; Neau et al. [4] and Dequatre-Ponchell et al. [11]: Fluindione, acénocoumarol and Warfarin)

**Table 2:** Risk of bias assessment according to the Cochrane ‘Tool to Assess Risk of Bias in Cohort Studies”

	1	2	3	4	5	6	7	8
Radberg et al. [5]	+ +	+	+	--	+ +	+ +	−	+ +
Neau et al. [4]	+	+ +	+	−	−	+ +	+	+
Rosand et al. [8]	+ +	+ +	+ +	+ +	+ +	+ +	+ +	+
Flibotte et al. [7]	+ +	+ +	+ +	+ +	+ +	+ +	+ +	+
Flaherty et al. [6]	+	+	+ +	+ +	+ +	+ +	+	+
Flaherty et al. [12]	+	+ +	+	−	+	+	−	+
Cucchiara et al. [29]	+	+ +	+ +	+ +	+ +	+ +	−	+
Horstmann et al. [9]	+ +	+	+	+	+	+ +	+	+
Ma et al. [21]	+ +	+ +	+ +	+ +	+ +	+	--	+
Dequatre-Ponchelle et al. [11]	+ +	+ +	+ +	–	+	+ +	--	+
Curtze et al. [20]	+ +	+ +	+ +	+	+	+ +	+ +	+
Von der Brelie et al. [10]	+ +	+	+ +	+	+	+ +	+ +	+ +
Inohara et al. [25]	+ +	+ +	+ +	+ +	+	+ +	+	+
Romem [27]	+ +	+ +	+ +	+ +	+	+	+	+
Roquer [23]	+ +	–	–	+	+	+	–	+
Toyoda [28]	+	+ +	+ +	+ +	+	+ +	+	+
Fric-Shamji [22]	+ +	+	+ +	+	+	+ +	+	+
Foerch [26]	+ +	+	+ +	+	+	+	--	+
Yamashita et al. [24]	+ +	+ +	+ +	+	–	+	+	+

1. Was selection of exposed and non-exposed cohorts drawn from the same population?

2. Can we be confident in the assessment of exposure?

3. Can we be confident that the outcome of interest was not present at start of study?

4. Did the study match exposed and unexposed for all variables that are associated with the outcome of interest or did the statistical analysis adjust for these prognostic variables?

5. Can we be confident in the assessment of the presence or absence of prognostic factors?

6. Can we be confident in the assessment of outcome?

7. Was the follow-up of cohorts adequate?

8. Were co-interventions similar between groups?

Ratings: +  + , definitely yes (Low risk of bias); + , probably yes; –, probably no; --, definitely no (high risk of bias)

### VKA-ICH vs. non-OAC ICH

#### Overview

All 19 studies reported data of 16,546 patients with VKA-ICH. The number of patients with VKA-ICH ranged between 21 and 208 patients in single- and multi-centre studies, with the GWTG registry reporting data of 15,036 patients with VKA-ICH. Fourteen of 19 studies recruited less than 100 patients with VKA-ICH. Patients with VKA-ICH were significantly older than patients with non-OAC ICH (mean age difference: 5.55 years, 95%CI 4.03–7.07, *p* < 0.0001, *I*^2^ = 92%, *p* < 0.001, supplemental figure).

#### Haematoma volume

**Fig. 1:**
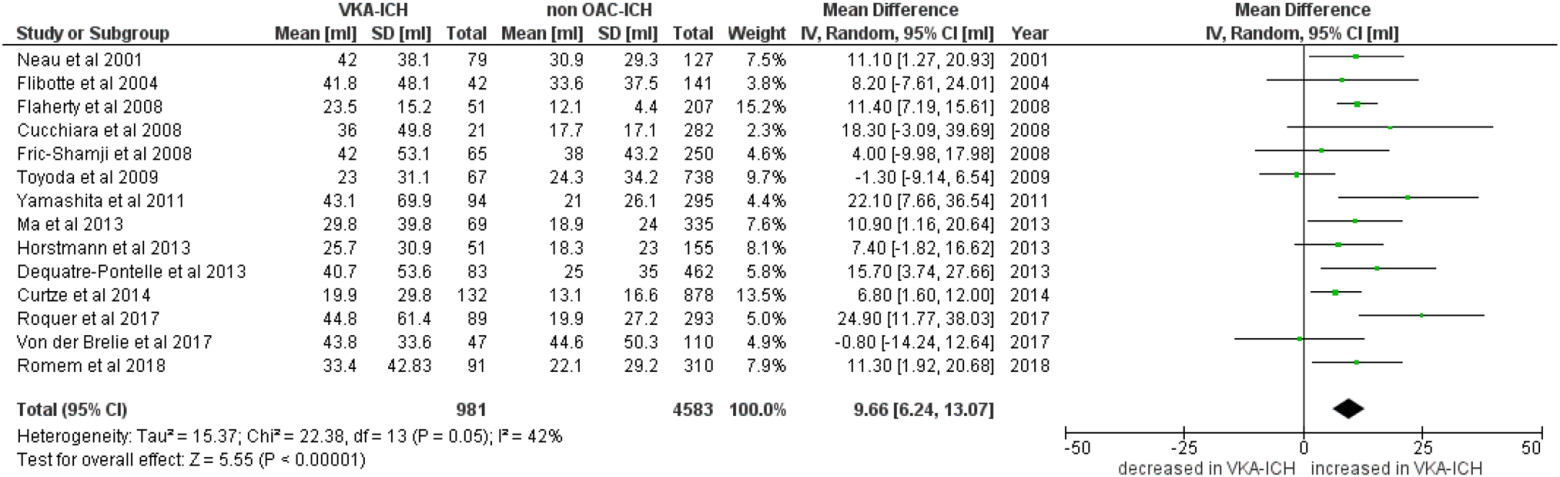
Mean difference of ICH volume in VKA-ICH compared to non-OAC ICH

Fourteen studies [4, 7, 9–12, 20–24, 27–29] reported data on ICH volume in 981 patients with VKA-ICH compared to 4583 patients with non-OAC ICH. In 7 studies, haematoma volume was measured using the ABC/2 formula [9, 11, 12, 20, 24], three studies used a planimetric software (ALICE or Analyze 10.0) [7, 21, 29]. Specifications of the used imaging techniques were provided in four studies [9–12, 24]. Details on applied methods considering ICH volume measurement are provided in the supplementary table. The mean ICH volume ranged from 19.9 ml to 44.8 ml in patients with VKA-ICH compared to 13.1 ml to 44.6 ml in non-OAC ICH. VKA-ICH was associated with significantly larger haematoma volume with a pooled mean volume difference of 9.66 ml (95%CI 6.24–13.07 ml, *p* < 0.00001; *I*^2^ = 42%, *p* = 0.05; Fig. 1).

#### Haematoma expansion

**Fig. 2:**
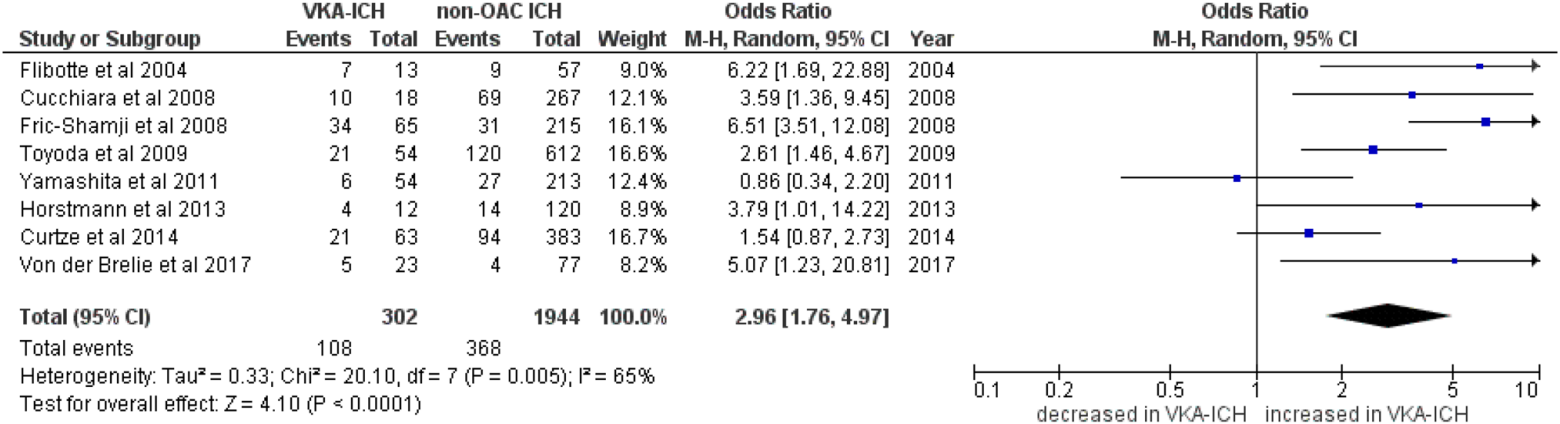
Rate of haematoma expansion (HE) in VKA-ICH compared to non-OAC ICH

Eight studies [7, 9, 10, 20, 22, 24, 28, 29] reported data on HE of 302 patients with VKA-ICH compared to 1944 patients with non-OAC ICH. Overall, VKA-ICH was associated with a significantly increased risk of HE (OR 2.96, 95%CI 1.74–4.97, *p* < 0.00001; *I*^2^ = 65%, *p* = 0.005, Fig. 2) with a pooled rate of 35.8% compared to 18.9% in patients with non-OAC ICH.

#### Mortality

**Fig. 3:**
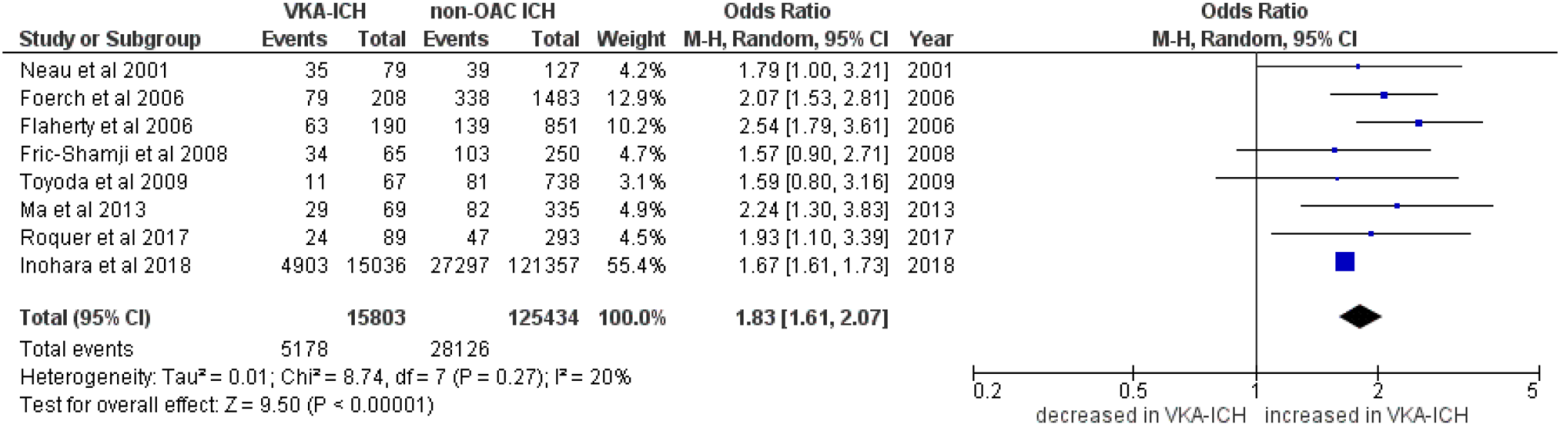
In-hospital mortality in VKA-ICH compared to non-OAC ICH

**Fig. 4:**
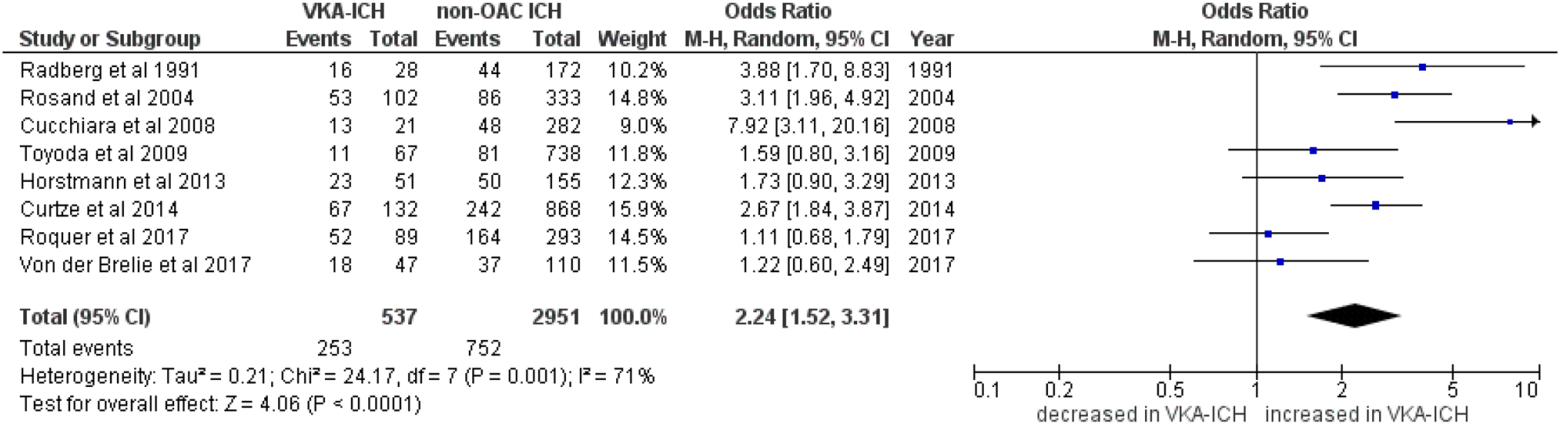
3-month mortality in VKA-ICH compared to non-OAC ICH

Fifteen studies reported mortality: eight studies [4, 6, 21–23, 25, 26, 28] reported in-hospital mortality in 15,803 VKA-ICH patients compared to 125,434 non-OAC ICH patients (of whom the majority were from the GWTG registry: 15,036 patients with VKA-ICH and 121,357 patients with non-OAC ICH). Eight studies [5, 8–10, 20, 23, 28, 29] reported 3-month mortality in 537 patients with VKA-ICH compared to 2951 patients with non-OAC ICH. VKA-ICH was associated with increased in-hospital mortality (Fig. 3; VKA-ICH: 32.8% vs. non-OAC ICH: 22.4%; OR 1.83, 95%CI 1.61–2.07, *p* < 0.00001, *I*^2^ = 20%, *p* = 0.27) and 3-month mortality (Fig. 4; VKA-ICH: 47.1% vs. non-OAC ICH: 25.5%; OR 2.24, 95%CI 1.52–3.31, *p* < 0.00001) although the data showed significant heterogeneity (*I*^2^ = 71%, *p *= 0.001) and more recent studies (i.e. published since 2009 [9, 20, 28]) and those published 2017 [10, 23] from specialized stroke centres showed lower mortality rates than older studies [5, 8, 29].

#### NOAC-ICH vs. non-OAC ICH

Only 2 studies included patients with NOAC-ICH: one single-centre study [10] from a German tertiary hospital (25 NOAC-ICH patients) and the GWTG registry [25]. The latter reported only data on in-hospital mortality; thus there were insufficient studies to perform a meta-analysis.

## Discussion

This systematic review and meta-analysis provided the most precise available estimates on key prognostic factors and outcomes for OAC-ICH. Our main findings were that compared to non-OAC ICH, VKA-ICH is associated with: (1) a higher mean haematoma volume of about 10 ml; (2) nearly twice the risk of in-hospital and 3-month mortality; and (3) nearly triple the risk of HE. There are insufficient data to draw firm conclusions about NOAC-ICH compared to non-OAC ICH.

This meta-analysis confirms, refines and expands current knowledge on the effect of OAC on ICH derived from prior studies. Several studies [4, 5, 12] described larger haematoma volumes in patients with VKA-ICH while more recent studies found no differences [9, 10] or differences only in subgroups of patients [11, 20]. Our meta-analysis has clarified and expanded these findings. We found that VKA-ICH is associated with significantly larger haematoma volumes. The increase of nearly 10 ml is clinically significant as haematoma volume is one of the most important predictors of poor outcome [30]; indeed, a haematoma volume of ≥ 30 ml has been included in the ICH-score [31] to predict outcome in ICH.

HE is also an important predictor of poor outcome and potential target for treatment [32, 33]. In the presence of OAC, it is feared that continuous bleeding leads to more and significant HE and rapid reversal of the anticoagulant effect is a widely accepted treatment priority [34–38]. The first study [7] reporting increased HE rates found a large difference between VKA-ICH (54%) and non-OAC ICH (16%). Our meta-analysis confirmed a significantly higher rate of HE but our more precise estimate suggests that the difference is much smaller than initially reported, with HE in 36% of patients with VKA-ICH and 19% in patients with non-OAC ICH. Nevertheless, we found heterogeneity among studies included in our meta-analysis, notably differences between studies from North America [7, 22, 29] reporting data from the early 2000s and studies from Europe and Asia [9, 10, 20, 24, 28] reporting more recent data. Regional differences in anticoagulation reversal strategies although not systematically reported in all studies might have played a role. European centres tend to use PCC combined with FFP and Vitamin K as first-line therapy for VKA reversal [35, 36, 38, 39] while in the US, FFP combined with Vitamin K has been the mainstay [7, 36, 37]. A recent randomized controlled trial found that PCC is superior to FFP in rapidly reversing VKA-related clotting abnormalities, but this study was underpowered to find differences in clinical outcomes [40]. The HE rate of 36% in patients with VKA-ICH is in line with the results from studies comparing HE between VKA-ICH and NOAC-ICH, which reported HE rates between 34% [16] and 37% [17] for patients with VKA-ICH. Another study investigating treatment strategies for VKA-ICH reported an overall HE rate of 36% in VKA-ICH [35]. Interestingly, the latter study found a HE rate of only 18% in VKA-ICH patients receiving most aggressive treatment (INR < 1.3 within 1 h and systolic blood pressure < 160 mmHg within 4 h) which is about the same rate as we found in non-OAC ICH (19%). This might point towards important treatment effects in VKA-ICH, suggesting potential normalization of HE rates if VKA-ICH is treated aggressively.

Although we found increased mortality in patients with VKA-ICH confirming prior research [4, 5, 8], there was significant heterogeneity among the published studies. Studies reporting data from the early 2000s or 1990s had worse outcome while more recent studies had less-pronounced differences. This might in part be explained by recent developments in acute blood pressure treatment (following INTERACT-2 [41] published in 2013) and widespread introduction of stroke unit care for patients with ICH [42]. Patients with VKA-ICH may particularly benefit from rapid and aggressive therapies in multi-disciplinary dedicated stroke unit teams. An aforementioned study [35] investigating treatment effects in VKA-ICH found the overall in-hospital mortality in VKA-ICH to be 31% which is in line with the findings of our meta-analysis (32.8%). Furthermore, in this study, patients receiving rapid blood pressure control and anticoagulation reversal had in-hospital mortality as low as only 13%, even lower as the 22% that we found in the non-OAC ICH group of our meta-analysis. Taken together, although our meta-analysis found HE and mortality to be higher in VKA-ICH, the heterogeneity of data included in our meta-analysis together with data from other studies might indicate that intensive treatment can significantly mitigate excessive HE and mortality reducing it to a level comparable to that of non-OAC ICH.

While many studies investigated differences between VKA-ICH and NOAC-ICH [13–17], we identified only 2 studies comparing NOAC-ICH with non-OAC ICH. Data from the US GWTG registry [25] found an increased in-hospital mortality but did not report data on haematoma volume, HE or 3-month mortality. The study from Von der Brelie et al. [10] found increased haematoma volume, HE and 3-month mortality in NOAC-ICH but this study included only 25 patients with NOAC-ICH.

Our study has several strengths: 1) combining and pooling data from multiple, independent studies with a large number of patients with VKA-ICH, we overcome limitations from prior smaller studies, refining and expanding their findings; 2) combining data from older with more recent studies allowed to highlight possible improvements in treatment of VKA-ICH underlining that further efforts to improve treatment are still important; 3) independent literature research and extraction of study data from two investigators reduced bias and increased data validity.

Our study has the following limitations: (1) we used aggregate observational data which are always prone to bias; (2) we were not able to perform multivariate analysis accounting for other potential predictors of haematoma volume, HE and mortality including age (VKA-ICH patients were 5 years older), acute treatment (blood pressure control and anticoagulation reversal) and other factors (e.g. comorbidities); (3) we were not able to calculate gender- or age-specific results. Furthermore, we did not have access to INR results at presentation. (4) Different methods applied to assess haematoma volumes have certainly contributed to the observed heterogeneity of results and are a source of bias. However, these methodological limitations applies to both, OAC-ICH and non-OAC-ICH. (5) Even with sophisticated measuring techniques, for large haematomas, the magnitude of haematoma volume measurement error may exceed the threshold used to dichotomize HE in the respective study [43]. In our analysis of aggregate data, we were not able to correct for this incertitude.

To summarize, this meta-analysis provided precise estimates of key prognostic variables and outcomes: we showed that VKA-ICH has larger haematoma volume, more frequent HE and higher mortality than non-VKA-ICH. Studies comparing NOAC-ICH and non-OAC ICH are scarce and current knowledge on NOAC-ICH is only available in relation to the effect of VKA-ICH. Further research on the effect of NOAC on these outcomes is needed.

## Electronic supplementary material

Below is the link to the electronic supplementary material.

Supplementary file1 (DOCX 34 kb)

Supplementary file2 (PNG 96 kb)

Supplementary file3 (PNG 17 kb)
