# Supplementary material for: Meta-analysis of haematoma volume, haematoma expansion and mortality in intracerebral haemorrhage associated with oral anticoagulant use
Source: J Neurol. 2019 Sep 20;266(12):3126–35. doi: 10.1007/s00415-019-09536-1 (PMC6851029; doi:10.1007/s00415-019-09536-1)
Supplement: Supplementary file 1 — Supplementary file1 (DOCX 34 kb) [file 415_2019_9536_MOESM1_ESM.docx]

**Supplemental material:**

Supplemental figure: Mean age difference between VKA-ICH and non-OAC ICH patients


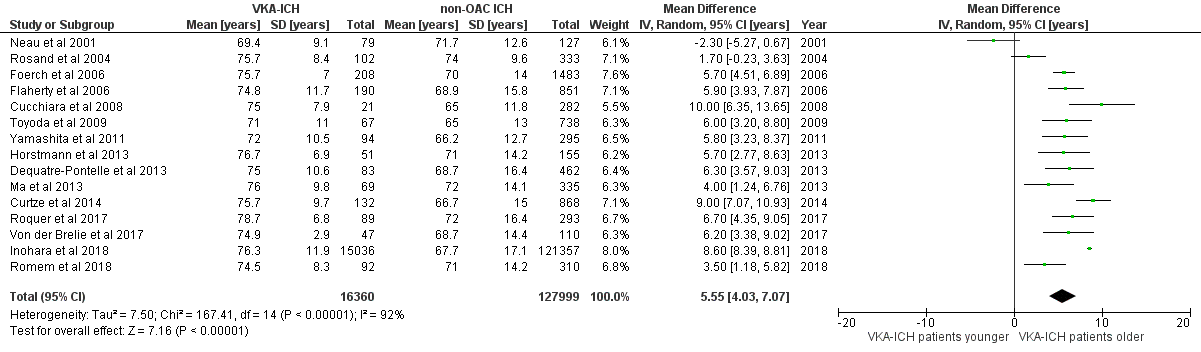


Supplemental table: Methods for assessment of haematoma volume

| Study | Year | ICH volume measurement method | MRI for volume measurement | | CT for volume measurement | | correction for uncertainty in volume measurements? |
| --- | --- | --- | --- | --- | --- | --- | --- |
|  |  |  | slice thickness | timepoint | slice thickness | timepoint |  |
| Neau | 2001 | ABC/2 |  |  |  | Not specified |  |
| Flibotte | 2004 | Analysis with Alice software,  IVH separately reported |  |  |  | all CT scans obtained within 7 days after admission | - exclusion of patients with brainstem and cerebellar ICH. - To assess a possible measurement drift, patients with volume reduction >/= 33% compared to baseline were also identified (3 patients = 4%) |
| Flaherty | 2008 | ABC/2, IVH excluded |  | first scan available (MRI or CT) |  | first scan available (MRI or CT) | - imaging analysis by two authors. testing for intraclass correlation of two reviewers (0.97 in 31 scans). - Log transformation to approximate normal distribution for statistical testng. |
| Cucchiara | 2008 | semiautomated planimetry and reconstruction with ALICE software, IVH reported |  | baseline scan (MRI or CT) |  | baseline scan (MRI or CT) | review by central neuroradiologist |
| Fric-Shamji | 2008 |  |  |  |  | admision CT scan |  |
| Toyoda | 2009 | ABC/2 |  |  |  | Admission CT scan | Volume assessment blinded to clinical history |
| Yamashita | 2011 | ABC/2 |  |  | 8-10mm, image matrix 340x340 | within 24 hours of onset | CT raters blind to clinical information |
| Ma | 2013 | Planimetric volume calculation with Analyze 10.0 software (Mayo clinic, Rochester), ICH and IVH separately, outline by tracing tools |  |  |  | baseline CT scan | review by two neurologists blinded to clinical status |
| Horstmann | 2013 | ABC/2 | 3 T MRI | baseline | 16 section CT scanner | baseline |  |
| Dequatre-Ponchelle | 2013 | ABC/2 |  |  | continuous slices, no gap, posterior fossa 3mm, hemispheres 5mm slice thickness | admission CT scan | imaging review by an investigator blinded to clinical data |
| Curtze | 2014 | ABC/2 |  | all subsequent scans after ICH |  | all subsequent scans after ICH | evaluation by neuroradiologists |
| Roquer | 2017 |  |  |  |  |  |  |
| Von der Brelie | 2017 | maximum axial diameter x maximum perpendicular diameter x number of axial CT scan layers x layer thickness/2 |  |  | 64 slice | initial CT scan |  |
| Romem | 2018 |  |  |  |  | CT scan |  |
