# Supplementary figures and images for: Meta-analysis of haematoma volume, haematoma expansion and mortality in intracerebral haemorrhage associated with oral anticoagulant use
Source: J Neurol. 2019 Sep 20;266(12):3126–35. doi: 10.1007/s00415-019-09536-1 (PMC6851029; doi:10.1007/s00415-019-09536-1)

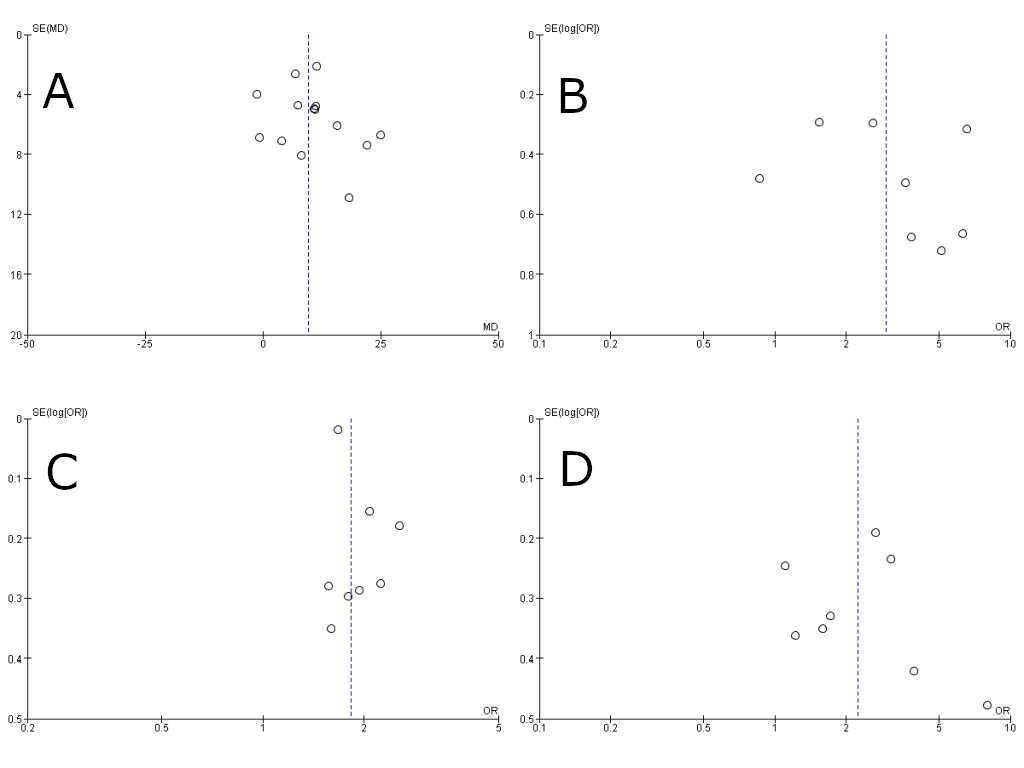

Supplement: Supplementary file 2 — Supplementary file2 (PNG 96 kb) [file 415_2019_9536_MOESM2_ESM.png]

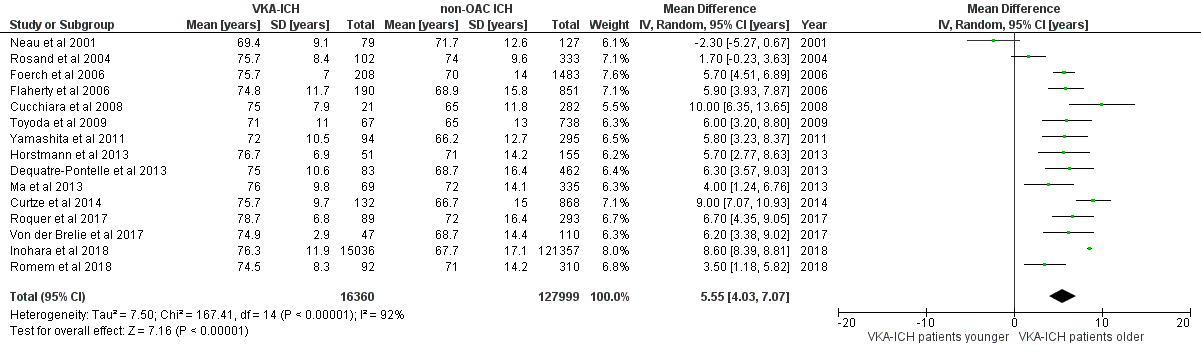

Supplement: Supplementary file 3 — Supplementary file3 (PNG 17 kb) [file 415_2019_9536_MOESM3_ESM.png]
